# Tuberculosis Chemotherapy Outcome in the Littoral Region of Cameroon: A Meta-analysis of Treatment Success Rate between 2014 and 2016

**DOI:** 10.1155/2020/8298291

**Published:** 2020-07-08

**Authors:** Dorgelesse F. Kouemo Motse, Dickson Shey Nsagha, Dieudonné Adiogo, Loick P. Kojom Foko, Pride M. Teyim, Alain Chichom-Mefire, Jules C. Nguedia Assob

**Affiliations:** ^1^Department of Medical Laboratory Sciences, Faculty of Health Sciences, University of Buea, Buea, P.O. Box 63, Buea, Cameroon; ^2^Department of Public Health and Hygiene, Faculty of Health Sciences, University of Buea, Buea, P.O. Box 63, Buea, Cameroon; ^3^Department of Biological Sciences, Faculty of Medicine and Pharmaceutical Sciences, University of Douala, P.O. Box 24157, Douala, Cameroon; ^4^Department of Animal Sciences, Faculty of Science, The University of Douala, P.O. Box 24157, Douala, Cameroon; ^5^NIMR-Indian Council of Medical Research (ICMR), Sector 8, Dwarka, 110077 New Delhi, India; ^6^Department of Microbiology and Parasitology, Faculty of Sciences, University of Buea, P.O. Box 63, Buea, Cameroon; ^7^Department of Surgery and Specialties, Faculty of Health Sciences, University of Buea, P.O. Box 63, Buea, Cameroon

## Abstract

**Background:**

Tuberculosis (TB) is a public health concern, especially in resource-constrained countries like Cameroon. TB drug resistance is a major obstacle to control and prevent.

**Design:**

Data from 2014 to 2016 on the outcome of anti-TB treatment in the Littoral Region were reviewed manually and analysed using the meta-analysis concept. The treatment success rates (TSR) were the primary outcome used for this study. The heterogeneity statistics (*I*^2^) was computed to orientate the choice of the best statistical model (binary fixed effect or random) to compute pooled value of TSR.

**Results:**

Using an intention-to-treat analysis, the pooled proportions of HIV-uninfected TB patients successfully cured from TB were low and slightly decreased by 1% between 2014 and 2016. Regarding HIV-infected TB patients, pooled values of TSR were lower than those of their HIV-negative counterparts with values ranging from 71% (95% CI: 63%-83%; *I*^2^ = 71.16%) in 2014 to 68% (95% CI: 58%-79%; *I*^2^ = 70.97%) in 2016. In addition, no heterogeneity was found in three years (*I*^2^ = 0.0%; *P* value = 1). These cure rates were strongly and negatively correlated with the rates of patients lost to follow-up regardless of the year. In HIV-infected patients, the pooled values of ITT analysis-based treatment success rates were 73% (*χ*^2^ = 13.92, *P* value = 0.0002), 71% (*χ*^2^ = 7.26, *P* value = 0.007), and 68% (*χ*^2^ = 8.02, *P* value = 0.004), respectively. The coverage rates with cotrimoxazole (CTX) gradually increased over year ranging from 78.90% in 2014 to 94.17% in 2016, similar to the coverage rate for ARV therapy that was 60.06% in 2014 against 90% in 2016. A positive and statistically significant correlation was found between the success of the anti-TB therapy in HIV-infected patients and coverage rates with CTX and ARV.

**Conclusion:**

An improvement in the reduction of percentage of lost to follow-up and coverage with CTX and ARV therapy could greatly increase chances to efficiently control TB in Cameroon.

## 1. Introduction

Infectious diseases represent a public health challenge especially in resource-limited countries. Tuberculosis (TB) is caused in humans by a bacterial pathogen belonging to genus *Mycobacterium* with *M. tuberculosis* as the main species involved in human cases and deaths. In 2018, TB was responsible for 10 million disease cases, 1.2 million deaths among HIV-negative, and 251,000 deaths among HIV-positive people, mainly in adults aged ≥15 years [1]. In Cameroon, TB is also a cause of concern with an incidence and a mortality rate of 194 and 30 for 100,000 people, respectively, with a predominance among females [1].

Since the achievement of full coverage of TB in Cameroon in 2002, the National Control Programme of TB has continuously improved its fight through policy and many key strategies including the implementation of surveillance system, a faster management through 248 diagnostic and treatment centers (DTCs) distributed over the territory. Also, there was an increase in full adherence to treatment as well as the proportion of cured patients through the adoption of directly observed treatment strategy (DOTS) based on rifampicin used as first-line treatment [2–4]. Recently, the National Control Programme validated the utilization of genotyping technology in the Littoral Region referred to as GeneXpert TB test for the detection of resistance to rifampicin [5]. However, tuberculosis still remains one of the public health priorities alongside malaria and HIV in Cameroon especially in the Littoral Region. People living in this region are highly at risk of suffering and dying from TB. This is supported by the fact that the Littoral Region is ranked third among the 10 regions of the country in terms of notification rates of new bacteriologically confirmed pulmonary tuberculosis [6]. In addition, HIV infection is also prevalent in the Littoral Region and complicates management of TB-infected patients like in most sub-Saharan Africa (SSA) countries. As a consequence, death rates in coinfected HIV/TB patients from SSA countries have risen over the last few decades [1].

We present in this study an overview of treatment outcomes of TB in Cameroon for the period 2014–2016 using a more robust analysis method based on meta-analysis approach. In addition, we evaluate the influence of loss to follow-up and death rates and the cotrimoxazole (CTX) and antiretroviral therapy (ART) coverage in treatment outcomes of the TB therapy by performing subgroup analysis. It is hoped that this overview will improve the understanding of the dynamics of TB treatment in Cameroon and inform policy.

## 2. Materials and Methods

### 2.1. Sources and Nature of Collected Data

Data of interest was obtained from the electronic database of the Regional Health Delegation in the Littoral Region upon obtaining all ethical clearance from the Institutional Review Board of the Faculty of Health Sciences of the University of Buea and administrative authorizations from regional delegation of public health for the Littoral Region. They were collected from 15 diagnostic and treatment centers (DTCs) of the Littoral Region, namely, CSI Delangue + Prison (CSIDelP), Dibamba Medical Center (DiMedC), Ekol-Mbeng CSI (EMCSI), Loum District Hospital (LDH), Mbanga District Hospital (MDH), St Jean de Malthe Hospital (StJDMH), CEBEC Ndoungue Hospital (CNH), Nkondjock District Hospital (NDH), Nkongsamba Protestant Hospital (NPH), Pouma Catholic Hospital (PCH), Epec Sakbayeme Hospital (ESH), Yabassi District Hospital (YDH), Melong District Hospital (MeDH), Alucam Medical and Social Center (AMSC), and Edea Regional Hospital (ERH).

Data collected consisted of the following: total number of TB-infected people treated, the number of people who successfully responded to treatment, the number of therapeutic failure (TF), the type of patients (HIV-uninfected and HIV-infected), and the number of HIV-positive people under cotrimoxazole (CTX) and antiretroviral therapy (ARV). These data were presented with respect to the above-mentioned DTCs. It is worth noticing that multidrug-resistant TB (MDR-TB) patients are not managed in these DTCs; thus, this category of patients was not included in the present study.

### 2.2. Outcomes of Interest

The treatment success rates (TSR) were the parameter investigated in the study. Treatment success rate corresponds to percentage of TB patients (new and relapse cases) registered under DOTS in a given year that successfully completed treatment, with or without bacteriological evidence of success (“cured” and “treatment completed,” respectively) [7, 8].

These parameters were computed using two approaches, namely, intention-to-treat (ITT) analysis and per protocol (PP) analysis. In the ITT analysis, the denominator is the total number of patients initially enrolled in a given year while in the PP analysis, the denominator is the total number of patients in whom the efficacy of treatment can be evaluated at the end of follow-up (i.e., success or failure). Thus, deaths, lost to follow-up, defaulted, and transferred out remain included in the ITT analysis while they are excluded from the calculation in PP analysis [9].

### 2.3. Data Management and Analysis

The manually extracted data was keyed into Excel and exported to the OpenMeta Analyst and SPSS version 25 software for meta-analysis and inferential statistics. Data was presented as charts or tables where appropriate. Spearman correlation and chi-squared tests were used to study both quantitative and qualitative variables, respectively. The significance was set at *P* value less than 0.05. The results from the meta-analysis were presented graphically using forest plots. We compute heterogeneity or *I*^2^ statistics, using the chi-squared test based on Cochran's *Q* statistic [10], to appraise the level of heterogeneity between DTCs included in the meta-analysis and choose the best statistical model (i.e., binary fixed effect or random effects models) to compute pooled value of TS and TF. Fixed effects model was suitable when *I*^2^ was ≤50%, while random effect model was suitable when *I*^2^ was ≥50% [11, 12]. The variance of individual DTCs was stabilized using the arcsine transformation of Freeman-Tukey prior to pooling estimates of proportion. Given the crucial role of CTX and ARV therapy in the efficacy of anti-TB therapy in HIV-infected patients, we evaluated the impact of CTX and ARV therapy coverage rates on TS using correlation test.

### 2.4. Ethical Clearance

This was obtained from the Institutional Review Board of the Faculty of Health Sciences of the University of Buea in Cameroon.

## 3. Results

### 3.1. Characteristics of Tuberculosis Patients

**Figure 1:**
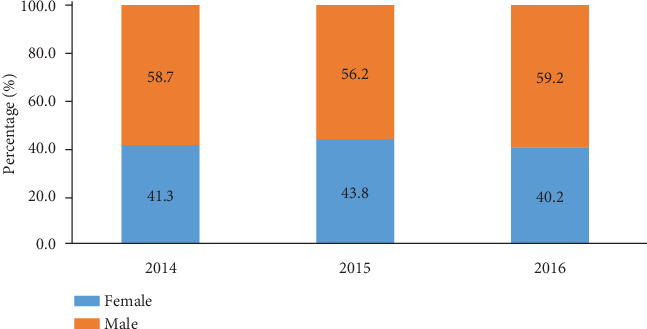
Distribution of patients with tuberculosis according to gender from 2014 to 2016.

**Figure 2:**
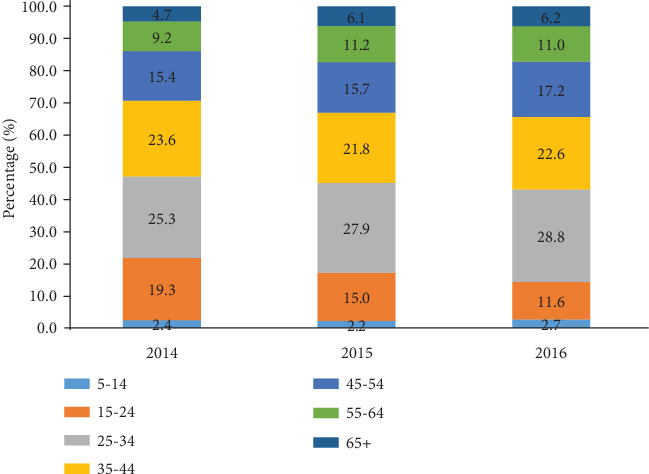
Distribution of patients with tuberculosis according to age from 2014 to 2016 in the Littoral Region of Cameroon.

The distribution of population with regard to gender and age is presented in Figure 1. Males accounted for majority of tuberculosis patients irrespective of the year, 58.7% in 20014, 56.2% in 2015, and 59.8% in 2016 (Figure 1). The population consisted mainly of individuals aged 25-34 years and 35-44 years (Figure 2). Children and adolescents (5-14 years) constituted 2.4%, 2.2%, and 2.7%, respectively, in 2014, 2015, and 2016.

### 3.2. Patients Registered in the National Control Programme in the Littoral Region

A total of 581, 501, and 441 TB-infected and HIV-uninfected individuals were registered in the National Control Programme in the Littoral Region in the years 2014, 2015, and 2016, respectively. Four DTCs, namely, CSIDelP, StJDMH, NPH, and LDH, managed more than half of registered patients irrespective of the year.

The prevalence of HIV infection among TB-positive patients has decreased over three years, from 38.50% in 2014 to 34.60% in 2016, even though none statistically significant difference was found (*χ*^2^ = 2.57; df = 2; *P* value = 0.726). Besides, a low level of heterogeneity (*I*^2^ = 21.86%; 95% CI: 0.00 to 97.38%; *P* value = 0.278) was found in the prevalence of HIV infection over years. Thus, the pooled prevalence of HIV infection was 36.47% (95% CI: 34.52% to 38.44%) based on fixed effects model. HIV-infected individuals were mainly recorded in the above-mentioned DTCs as well. The rate of coverage with both cotrimoxazole and antiretroviral (ARV) therapy gradually increased from 2014 to 2016. For cotrimoxazole, the coverage ranged from 70.98% in 2014 to 94.17% in 2016, and for ARV therapy, it was 60.06% in 2014 against 90% in 2016 (Table 1).

### 3.3. Trends of Anti-TB Treatment Success over Time in HIV-Negative Individuals

**Figure 3:**
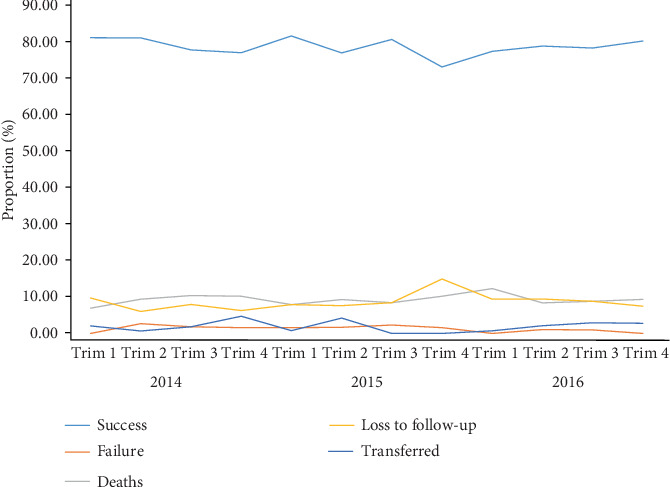
Evolution of proportion of treatment success, treatment failure, deaths, loss to follow-up, and transfer in HIV-uninfected patients with regard to trimester (Trim) and year (2014-2016) in the Littoral Region of Cameroon.

**Figure 4:**
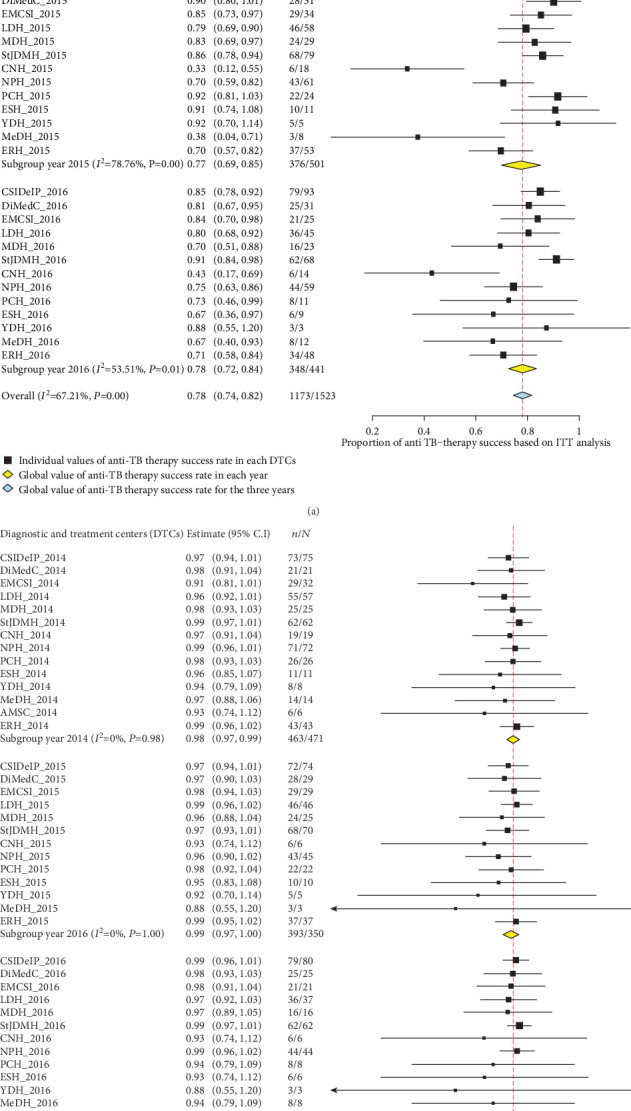
Forest plots of pooled values of anti-TB treatment success rate in HIV-uninfected patients with regard to year and DTCs using ITT analysis (a) and PP analysis (b) from 2014 to 2016 in the Littoral Region of Cameroon. TB: tuberculosis; *N*: total number; *n*: number of patients cured for TB; the horizontal lines through the black boxes depicts the length of confidence interval at 95%; TB: tuberculosis; DTCs: diagnostic and treatment centers; ITT: intention-to-treat; CSIDelP: CSI Delangue + Prison; DiMedC: Dibamba Medical Center; EMCSI: Ekol-Mbeng CSI; LDH: Loum District Hospital; MDH: Mbanga District Hospital; StJDMH: St Jean de Malthe Hospital; CNH: CEBEC Ndoungue Hospital; NDH: Nkondjock District Hospital; NPH: Nkongsamba Protestant Hospital; PCH: Pouma Catholic Hospital; ESH: Epec Sakbayeme Hospital; YDH: Yabassi District Hospital; MeDH: Melong District Hospital; AMSC: Alucam Medical and Social Center; ERH: Edea Regional Hospital.

The proportion of HIV-uninfected TB patients successfully cured from TB has slightly decreased between 2014 and 2016 (Figure 3). In addition, the pooled values calculated based on ITT analysis were 79% (95% CI: 73%-85%), 77% (95% CI: 69%-85%), and 78% (95% CI: 72%-84%), respectively, as depicted in the forest plot on Figure 4(a). The individual values of anti-TB treatment success rates, based on ITT analysis, were quite different from DTCs to another during three years as witnessed by high and statistically significant of heterogeneity (*I*^2^ = 63.55% in 2014; *I*^2^ = 78.76% in 2015; *I*^2^ = 53.51% in 2016; *P* value = 0.001), with an overall heterogeneity of 67.21% (Figure 4(a)). In contrast, using the PP analysis, the treatment rates were high (i.e., above 90%) and constant with a three-year pooled value of 98% (forest plot on Figure 4(b)). In addition, no heterogeneity was found in three years (*I*^2^ = 0.0%; *P* value = 1) (Figure 4(b)).

### 3.4. Trends of Anti-TB Treatment Success over Time in HIV-Positive Individuals

**Figure 5:**
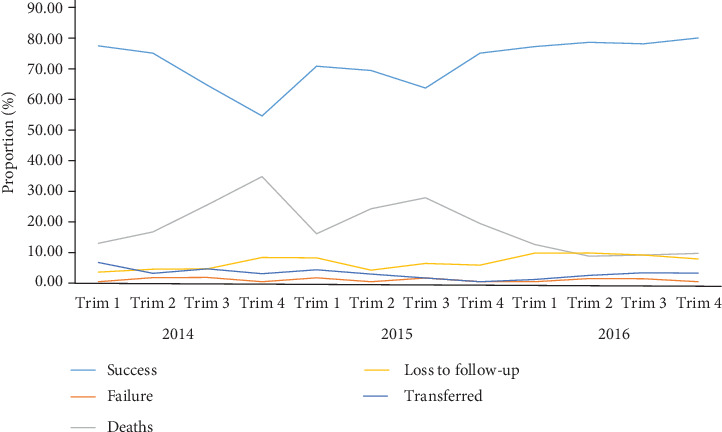
Evolution of proportion of treatment success, treatment failure, deaths, loss to follow-up, and transfer in HIV-infected patients with regard to trimester (Trim) and year (2014-2016) in the Littoral Region of Cameroon.

**Figure 6:**
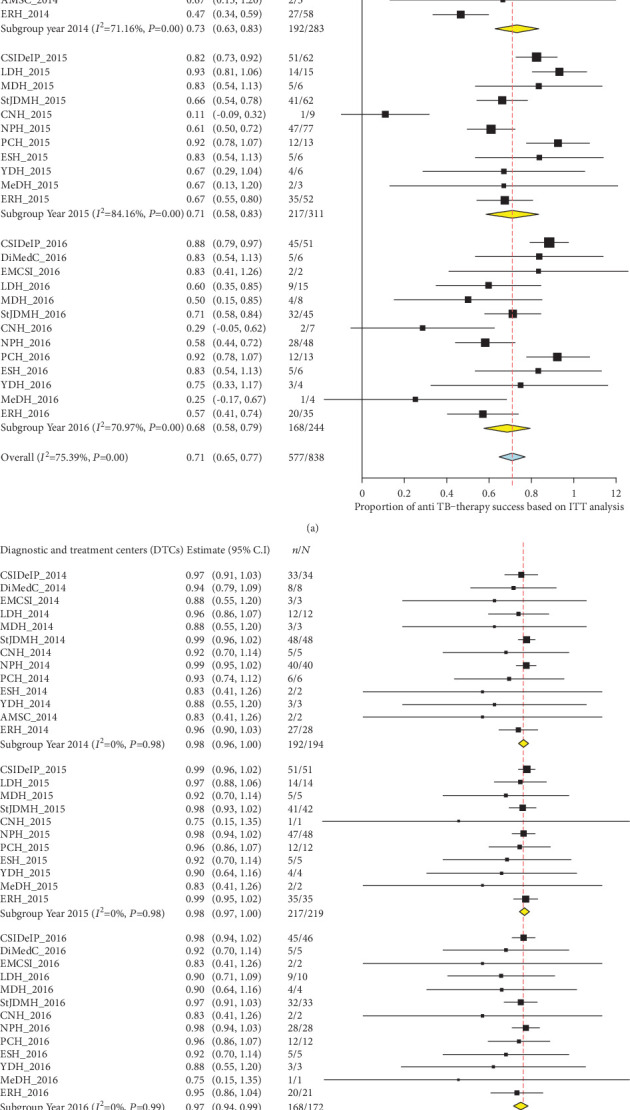
Forest plots of pooled values of anti-TB treatment success rate in HIV-positive patients with regard to year and DTCs using ITT analysis (a) and PP analysis (b) from 2014 to 2016 in the Littoral Region of Cameroon. : individual values of anti-TB therapy success rate in each DTCs; : global value of anti-TB therapy success rate in each year; : global value of anti-TB therapy success rate for the three years; TB: tuberculosis; *N*: total number; *n*: number of patients cured for TB; the horizontal lines through the black boxes depicts the length of confidence interval at 95%; TB: tuberculosis; DTCs: diagnostic and treatment centers; HIV: human immunodeficiency virus; ITT: intention-to-treat; CSIDelP: CSI Delangue + Prison; DiMedC: Dibamba Medical Center; EMCSI: Ekol-Mbeng CSI; LDH: Loum District Hospital; MDH: Mbanga District Hospital; StJDMH: St Jean de Malthe Hospital; CNH: CEBEC Ndoungue Hospital; NDH: Nkondjock District Hospital; NPH: Nkongsamba Protestant Hospital; PCH: Pouma Catholic Hospital; ESH: Epec Sakbayeme Hospital; YDH: Yabassi District Hospital; MeDH: Melong District Hospital; AMSC: Alucam Medical and Social Center; ERH: Edea Regional Hospital.

In 2014, the proportion of treatment success gradually decreased and then increased as from 2015 to 2016 (Figure 5). In contrast, the proportion of deaths showed an inverted trend to that of treatment success while the proportion of loss to follow-up, failure, and transfer remained stable over from 2014 to 2016. As regarding HIV-infected TB patients, the ITT analysis-based efficacy of anti-TB therapy gradually decreased over years as shown in the forest plot on Figure 6(a). A high and statistically significant heterogeneity (*I*^2^ = 71.16% in 2014; *I*^2^ = 84.16% in 2015; *I*^2^ = 70.97% in 2016; *P* value = 0.001) was found with regard to the year. Thus, the pooled value of treatment success based on random effect model was 73%, 71%, and 68%, respectively, in 2014, 2015, and 2016 (forest plot on Figure 6(a)). The majority of treatment failures were due to deaths. As found in HIV-uninfected individuals, the cure rates were close to 100% in the PP analysis in HIV-infected individuals (Figure 6(b)). In addition, it should be noted that the treatment success was significantly higher in HIV-uninfected individuals compared to their HIV-infected counterparts regardless of the year (2014: 79% versus 73%, *χ*^2^ = 13.92, *P* value = 0.0002; 2015: 77% versus 71%, *χ*^2^ = 7.26, *P* value = 0.007; and 2016: 78% versus 68%, *χ*^2^ = 8.02, *P* value = 0.004).

### 3.5. Impact of Loss to Follow-Up on the Success of the Anti-TB Therapy in HIV-Negative Patients

The treatment success rates were strongly and negatively correlated with the rates of patients lost to follow-up regardless to the year. The correlation coefficient values were statistically significant: *r* = −0.67 and *P* value = 0.007 for 2014, *r* = −0.62 and *P* value = 0.022 for 2015, and *r* = −0.80 and *P* value = 0.0006 for 2016. Also, negative correlations were found between percentage of TB treatment success and the percentage of death, but none of them were statistically significant.

### 3.6. Impact of Deaths Rates on the Success of the Anti-TB Therapy in HIV-Positive Patients

**Figure 7:**
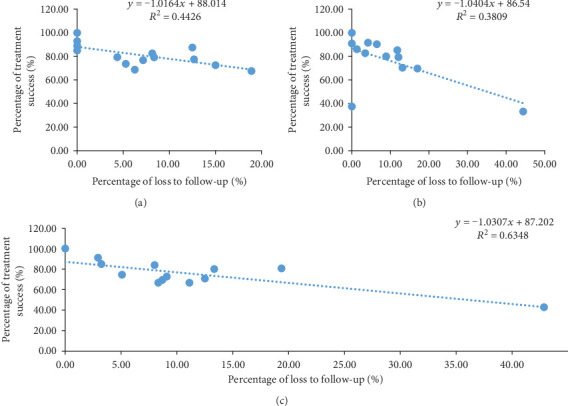
Correlation between the percentage of ITT-based treatment success in HIV-negative patients and the percentage of patients lost to follow-up in 2014 (a), 2015 (b), and 2016 (c) in the Littoral Region of Cameroon.

**Figure 8:**
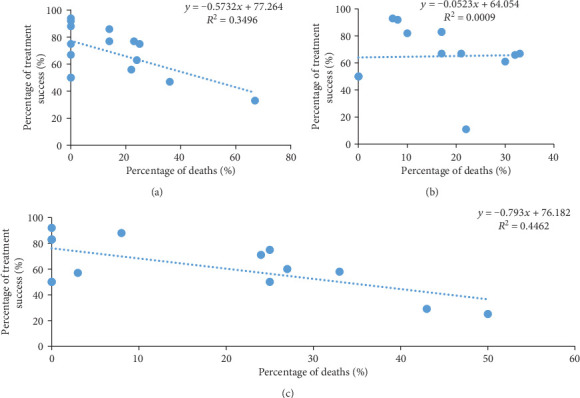
Correlation between the percentage of ITT-based treatment success in HIV-positive patients and the percentage of deaths in 2014 (a), 2015 (b) and 2016 (c) in the Littoral Region of Cameroon.

The treatment success rates were strongly and negatively correlated with the rates of deaths regardless to the year with the exception of 2015 (Figure 7). The correlation coefficient values were: *r* = −0.59 and *P* value = 0.0186 for 2014; *r* = 0.029 and *P* value = 0.919 for 2015 and *r* = −0.67 and *P* value = 0.005 for 2016. Negative correlations were found between percentage of TB treatment success and the percentage of lost to follow-up, but none of them were statistically significant. Similarly, negative correlations were found between the success rates and percentage of deaths in 2014 and 2016, unlike in 2015 where positive correlation was found (Figure 8).

### 3.7. Impact of Cotrimoxazole and Antiretroviral Therapy Coverage Rates on the Success of Anti-TB Therapy in HIV-Positive Patients

A positive and statistically significant correlation was found between the cotrimoxazole coverage rate and the treatment success in 2015 and 2016 (Table 2). As regards the link between the ARV coverage rate and the treatment success, we found a strong and statistically significant correlation in 2016 only (*r* = 0.806; *P* value = 0.0004).

## 4. Discussion

### 4.1. Cure Rates of TB in HIV-Infected and HIV-Uninfected TB Patients

Tuberculosis still remains one of the main causes for concern in Cameroon. We used a meta-analysis to compute the efficacy of anti-TB therapy over three years. The pooled values of percentage of cured patients, both HIV-uninfected and HIV-infected, have gradually decreased over time. This is partially attributable to the high rate of patients lost to follow-up as showed in this study. The findings are consistent with previous findings that outlined that nonadherence to treatment was the main cause of lost to follow-up [13]. Thus, it would be of utmost importance to develop and adopt other strategies to improve the adherence to anti-TB therapy. It was in this light that Bediang and colleagues explored the effect of SMS reminders in the treatment adherence and cure rates, though no improvement in these both rates were reported [14].

Besides, the pooled values of cure rates were below the threshold of 85% defined by the WHO [15]. The appearance and spread of multidrug-resistant strains can also be explained by this finding as they have been reported in the Littoral Region by many authors [4, 6, 16]. Also, this problem of multidrug resistance can become a bigger threat in the following years in the region due to high rates of cross-border and between-region migrations [17].

We used two approaches to compute the data and the pooled prevalence of treatment success, namely, ITT and PP analyses. Heterogeneity was higher in ITT analysis as compared to PP analysis, and this can be due to the fact that these two methods rely on different bases for calculating treatment success as above-mentioned. In PP analysis, denominator used to compute TSR corresponds to the number of people in whom the efficacy of therapy can be evaluated at the end of follow-up while the total number of initially enrolled patients constitutes the denominator in ITT analysis. ITT is considered as a standard for analysis of clinical trials due to the fact that its results reflect better clinical practice characterized by a high between-study heterogeneity. Thus, it is not surprising to have a high heterogeneity in TS between different DTCs using ITT analysis. Besides, ITT analysis allows for maintenance of comparability between groups, sample size, and eliminates bias [18, 19].

### 4.2. Impact of HIV Infection on TB Cure Rates

The treatment rates were significantly lower in TB/HIV-coinfected people using the ITT analysis, and this is consistent with previous hospital-based study conducted in Yaoundé [20]. The deaths accounted mainly for the treatment failure cases. These authors also reported high mortality rates among HIV/TB coinfected which was likely due to a higher risk of infection with opportunistic diseases, comorbidities, or other non-HIV-related disorders due to impaired immune response caused by HIV infection [20, 21]. TB is the commonest opportunistic infection associated with impaired health status and mortality in people living with HIV [22].

### 4.3. Impact of CTX and ARV Therapy on Anti-TB Therapy Success Rates

We found that not all HIV-infected individuals were under cotrimoxazole and under ARV therapy despite an increase in the coverage rate from 2014 to 2016. This was shown by a strong positive correlation between the rates of coverage with CTX and ARV therapy and anti-TB therapy success, thereby outlining the higher CTX or ARV therapy rates, the higher anti-TB therapy success rates. Also, the complication of HIV disease due to the absence of ARV therapy could also have been involved in some of these deaths. Cotrimoxazole (CTX) and ARV therapies are both necessary to reduce the risk of deaths in HIV/TB-coinfected people. Cotrimoxazole is an antibiotic used to prevent the occurrence of opportunistic diseases while the ARV therapy is used to slow down the course of the disease and stabilize the immune system of HIV patients [23, 24]. Our findings are consistent with those of previous studies in Cameroon [20, 25].

### 4.4. Limitations of the Study

The findings of this study should be interpreted in light of its limitations. First, the study analysed date from 15 DTCs of the Littoral Region, and this does not reflect the picture at the national level. Second, we found strong correlation between TSR with both CTX and ART therapy coverages in HIV-positive TB patients. However, we did not explore other factors, such as treatment adherence and intrinsic susceptibility of *M. tuberculosis* strains to anti-TB drugs for instance, that are also key determinants of drug therapy outcome [1].

In conclusion, this study outlined a slight decrease in the anti-TB treatment success rates from 2014 to 2016. These rates were lower than the WHO threshold in both TB infected alone and TB/HIV coinfected and were mainly attributable to lost to follow-up and nonobservance to CTX/ARV, respectively. Finally, this study also points out a need for strengthening the policies for cotrimoxazole and ARV therapy in TB/HIV coinfected. An improvement in the reduction of percentage of lost to follow-up and CTX and ARV therapy coverage could greatly increase the chances to efficiently control TB in Cameroon. There is need to develop, implement, or reinforce strategies aimed at limiting the magnitude of loss to follow-up and increasing the coverage rates with cotrimoxazole and antiretroviral therapy. These strategies should be focused mainly on change in behavior of TB patients.

## Figures and Tables

**Table 1:** Cotrimoxazole and antiretroviral drugs coverage among HIV-infected patients according to the DTC and year (2014-2016) in the Littoral Region of Cameroon.

	Cotrimoxazole coverage									Antiretroviral therapy coverage								
	Year 2014			Year 2015			Year 2016			Year 2014			Year 2015			Year 2016		
DTCs	*N*	*n*	%	*N*	*n*	%	*N*	*n*	%	*N*	*n*	%	*N*	*n*	%	*N*	*n*	%
CSIDelP	58	58	100.0	62	61	98.39	51	50	98.04	58	49	84.48	62	54	87.10	51	47	92.16
DiMedC	7	3	42.86	0	0	NA	6	4	66.67	7	0	0.00	0	0	NA	6	5	83.33
EMCSI	4	4	100.0	0	0	NA	2	2	100.0	4	3	75.0	0	0	NA	2	2	100.0
LDH	19	19	100.0	15	15	100.0	12	11	91.67	19	0	0.00	15	4	26.67	12	9	75.00
MDH	6	5	83.33	7	5	71.43	7	6	85.71	6	0	0.00	7	0	0.00	7	5	71.43
StJDMH	62	62	100.0	62	62	100.0	45	45	100.0	62	59	95.16	62	62	100.0	45	42	93.33
CNH	9	4	44.44	8	2	25.0	7	2	28.57	9	0	0.00	8	1	12.50	7	1	14.29
NDH	0	0	NA	0	0	NA	0	0	NA	0	0	NA	0	0	NA	0	0	NA
NPH	63	39	61.90	77	70	90.91	48	47	97.92	63	30	47.62	77	62	80.52	48	45	93.75
PCH	7	5	71.43	13	13	100.0	13	13	100.0	7	5	71.43	13	13	100.0	13	13	100.0
ESH	7	7	100.0	6	5	83.33	6	6	100.0	7	3	42.86	6	3	50.0	6	6	100.0
YDH	4	2	50.0	6	6	100.0	4	4	100.0	4	1	25.0	6	5	83.33	4	4	100.0
MeDH	2	2	100.0	3	3	100.0	4	1	25.0	2	2	100.0	3	2	66.67	4	2	50.0
AMSC	2	0	0.00	0	0	NA	0	0	NA	2	0	0.00	0	0	NA		0	NA
ERH	58	40	68.97	52	42	80.77	35	35	100.0	58	33	56.90	52	34	65.38	35	35	100.0
Total	308	243	78.90	311	284	91.32	240	226	94.17	308	185	60.06	311	240	77.17	240	216	90.00

*N*: total number; *n*: number of patients under ARV therapy; DTCs: diagnostic and treatment centers; HIV: human immunodeficiency virus; ITT: intention-to-treat; CSIDelP: CSI Delangue + Prison; DiMedC: Dibamba Medical Center; EMCSI: Ekol-Mbeng CSI; LDH: Loum District Hospital; MDH: Mbanga District Hospital; StJDMH: St Jean de Malthe Hospital; CNH: CEBEC Ndoungue Hospital; NDH: Nkondjock District Hospital; NPH: Nkongsamba Protestant Hospital; PCH: Pouma Catholic Hospital; ESH: Epec Sakbayeme Hospital; YDH: Yabassi District Hospital; MeDH: Melong District Hospital; AMSC: Alucam Medical and Social Center; ERH: Edea Regional Hospital.

**Table 2:** Correlation between the anti-TB therapy success and rate coverage of CTX and ARV in HIV-positive patients from 2014 to 2016 in the Littoral Region of Cameroon.

Nature of the correlation	*n*	*r*	95% CI	*P* value
Treatment success and CTX coverage rates				
Year 2014	14	-0.044	-0.562 to 0.498	0.883
Year 2015	11	0.779	0.337 to 0.940	0.003^∗^
Year 2016	13	0.758	0.355 to 0.923	0.001^∗^
Treatment success and ARV coverage rates				
Year 2014	14	-0.254	-0.692 to 0.319	0.388
Year 2015	11	0.240	-0.421 to 0.734	0.489
Year 2016	13	0.806	0.458 to 0.940	0.0004^∗^

TB: tuberculosis; CTX: cotrimoxazole; ARV: antiretroviral; HIV: human immunodeficiency virus; *n*: frequency; *r*: Spearman correlation coefficient; 95% CI: confidence interval at 95%; Spearman correlation test and significance was set at *P* value less than 0.05 (^∗^).

## Data Availability

The data used to support the findings of this study are available from the corresponding author upon request.
